# A Novel Auditory-Cognitive Training App for Delaying or Preventing the Onset of Dementia: Participatory Design With Stakeholders

**DOI:** 10.2196/19880

**Published:** 2020-09-30

**Authors:** Emily Frost, Talya Porat, Paresh Malhotra, Lorenzo Picinali

**Affiliations:** 1 Dyson School of Design Engineering Imperial College London London United Kingdom; 2 Division of Brain Sciences Imperial College London London United Kingdom

**Keywords:** cognitive decline, mobile phone, hearing loss

## Abstract

**Background:**

Multiple gaming apps exist under the dementia umbrella for skills such as navigation; however, an app to specifically investigate the role of hearing loss in the process of cognitive decline is yet to be designed. There is a demonstrable gap in the utilization of games to further the knowledge of the potential relationship between hearing loss and dementia.

**Objective:**

This study aims to identify the needs, facilitators, and barriers in designing a novel auditory-cognitive training gaming app.

**Methods:**

A participatory design approach was used to engage key stakeholders across audiology and cognitive disorder specialties. Two rounds, including paired semistructured interviews and focus groups, were completed and thematically analyzed.

**Results:**

A total of 18 stakeholders participated, and 6 themes were identified to inform the next stage of app development. These included congruence with hobbies, life getting in the way, motivational challenge, accessibility, addictive competition, and realism.

**Conclusions:**

The findings can now be implemented in the development of the app. The app will be evaluated against outcome measures of speech listening in noise, cognitive and attentional tasks, quality of life, and usability.

## Introduction

### Background Research

Globally, approximately 50 million people live with diagnosed dementia, with this figure expected to increase to 82 million in the next 10 years [1]. At present, no treatment is available to either cure or prevent dementia, which has led the World Health Organization (WHO) to classify dementia as a public health priority. The call to action is to reduce the risk of developing dementia through early diagnosis of cognitive decline, intervention, and eventually prevention.

A commission by Livingston et al [2] concluded that 35% of dementia diagnoses were potentially preventable and have identified 9 modifiable risk factors with the capability of preventing dementia. Of these 9 risk factors, midlife hearing loss was found to be the highest potentially modifiable factor at 9%. In comparison, other modifiable factors in later life included smoking (5%), depression (4%), and social isolation (2%). This evidence concurs with previous research, suggesting that age-related hearing loss (presbyacusis) increases the risk of developing dementia in later life by up to 5 times [3]. Despite these findings, the causality in this relationship is still unknown. Furthermore, whether any form of rehabilitation, either through hearing aids, auditory training, or assistive listening devices, could delay or prevent the onset of dementia symptoms is also unknown [2].

The most robust methodology to further investigate this relationship would be an adequately powered, longitudinal, randomized controlled trial. A complex study such as this would need to ensure that any treatments, such as hearing aids, were adhered to throughout the study. The adoption and use of hearing aids is relatively low. In the age bracket of people aged 55 to 74 years, 80% of people who require hearing aids do not have them [4]. This would be a key issue to address in the design of such a trial. Other methodologies should, also, be explored, and as hearing loss in midlife could be a preventable factor, the focus, as outlined by the WHO, should be on early detection and intervention. Cohort studies have suggested that presbyacusis tends to precede dementia onset by 5 to 10 years [5]. Interestingly, on average, people tend to wait for 10 years before they seek help for their hearing loss [6]. Hearing loss can be diagnosed with a simple and quick diagnostic test and is an easily measurable critical factor in potentially preventing cognitive impairment.

This study highlights an under-researched group of people who may be in the early stages of presbyacusis and present with a mild to moderate hearing loss, do not seek treatment but have an increased risk of developing dementia. There is potential to investigate these *preclinical symptoms* of dementia in this group by targeting the areas of the brain that contribute to auditory and cognitive functions, with the possibility of delaying the onset of these symptoms. This area of research has the potential to impact on what is likely to be one of the largest health care issues of the next century.

### Gamification in Dementia Research

One potentially more achievable alternative to a formal randomized controlled trial is to engage people in preventing dementia symptoms through gamification. Gamification has been shown to be an effective research tool that can demonstrate and maintain health behavior change [7]. A gamified app would be highly accessible within the home environment and less challenging than seeking general practitioner (GP) treatment for initial changes in cognition.

A literature review of games aiding early diagnosis of dementia, particularly Alzheimer disease (AD) [8], concluded that games could be utilized to overcome important barriers in the AD diagnosis process. Delays in self-referral, physician factors, age, and available services for assessing cognitive disorders were all identified as potential obstacles. A gaming app could be more motivational than a written memory assessment, maintaining a low cost/high reward ratio if evidence demonstrated that the appl could delay or prevent the onset of dementia symptoms.

Anguera et al [9] tested the hypothesis that playing the three-dimensional multitasking driving video game *NeuroRacer* could improve cognition that was previously diminished through healthy aging. Older adults (n=46) demonstrated less multitasking costs when compared with controls over a 4-week playing period, with effects sustained at *6-month follow-up. Of particular importance was the finding of a Transfer of Benefit.* The authors claim that by playing the driving video game, participants demonstrated improvements in both working memory and sustained attention—2 abilities that were not specifically targeted by the video game. This transfer of benefit outside of the on-task performance was a novel finding. The authors suggest possible reasoning for this being (1) the use of a video game outside of a typical laboratory environment and (2) the custom nature of the video game. As far as we are aware, there has been no attempt to address hearing loss and impaired speech perception using such an approach.

Another example is *Kitchen and Cooking* [10], which was designed and evaluated as a game to assess the executive function of planning. Different cooking recipes could be played by participants with mild cognitive impairment (MCI) and AD. Compared with the AD group (n=12), the MCI group (n=9) showed significant improvement in the Stroop test performance over a 4-week period. It is unclear from the results if this improvement would be sustained over a longer period of time, as this pilot study collected data for only 4 weeks across a small sample. However, the results lend support to the notion that interventions aimed at training cognitive abilities may be more effective in the predementia stage [11].

It is unclear whether the MCI group’s improvements compared with the AD group would have been any different from that of a healthy control group. There was a large variability in playing time within the small sample size. This not only emphasizes the importance of designing a game that is capable of engaging and maintaining interest but also focuses on the ability to measure levels of engagement and evaluate how different levels of engagement impact levels of effect. As suggested by Anguera et al [9], the success of *NeuroRacer* was attributed to the custom design of the game. *Kitchen and Cooking* was the premise for the design because food was rated as the most interesting area for older people in nursing homes. Thus, it would be prudent to employ a participatory design by involving key stakeholders in customizing the design of future games and evaluating the results with both validated quantitative measures and qualitative interviews.

One study used a qualitative methodology to investigate older adults’ perceptions of playing the Xbox Kinect game *Dr. Kawashima’s Brain Training* as a way of maintaining their cognition through intellectual exercise [12]. As previously suggested to ensure that a game is successfully adopted by the intended user group, the design should be appropriate to engage the specific population. Talaei-Khoei and Daniel [12] attribute this to a *perceived transfer effect.*

This occurs when adults who see a cognitive game as empowering, rather than supportive, which equate to a higher potential to yield long-term benefits. Rather, it is not only the content of the game but how participants view the content with respect to their own selves. A key finding was that the mini-games in *Dr. Kawashima’s Brain Training* were perceived to be useful in maintaining cognition and transference to real-world daily tasks, such as reading. Participants (n=21) felt that by sustaining functions through the mini-games they would be able to live independently longer providing a long-term transfer of benefit.

Other key findings distinguish the perceptions of supportive and empowering technologies. For instance, the use of hearing aids is categorized as supportive. Hearing instruments can only aid a person in a functional ability that has already begun to decline. This could lead to hearing aids being perceived as less useful, particularly in the long term. In contrast, an empowering virtual game focusing on active auditory training could be perceived as having transferable long-term effects on cognitive ability. The authors also concluded that more qualitative research was required in the field, especially on why end users would think a training game would be useful and adopt it.

The literature shows that gamification can provide a platform for customized, home-based training in different areas of health behavior change, including cognitive performance. Previous studies have demonstrated that certain games have the potential to transform the benefits of virtual play into self-confidence for maintaining cognitive effort for daily activities. More specifically, using training games that are deemed useful and engaging for users at the predementia stage may be more effective than after a dementia diagnosis. Given the findings from Livingston et al [2], there is a demonstrable gap in the use of games to investigate age-related hearing loss and cognitive decline. An iteratively designed app using qualitative inputs from key stakeholders to investigate the role of hearing loss and speech perception in cognitive impairment is yet to be developed. The use of participatory design with specific stakeholder engagement would have the ability to further investigate this area.

### Aims and Objectives

The overall aim of this study is to investigate whether an empowering gaming app can be designed to engage users in the midlife population at risk of presbyacusis and mild or subjective cognitive impairment to improve speech perception and cognitive performance.

This aim will be achieved with the following objectives:

- To adopt a participatory design approach with relevant stakeholders to produce an iteratively designed auditory-cognitive training app
- To understand the facilitators and barriers to producing an auditory-cognitive training app
- To identify the specific design requirements for an auditory-cognitive training app

## Methods

### Participants

A total of 18 relevant stakeholders (service users, clinicians, researchers) were recruited across audiology and cognitive disorder clinics at Imperial College Healthcare, across research groups at Imperial College London and their corresponding research networks. Participants were chosen using an opportunity sampling method as it was a convenient way of accessing clinical, service user, and researcher expertise. Participants were included in this study if they were considered to be a stakeholder and had the capacity to provide informed written consent. Professionals were considered key stakeholders by the research team if they had experience with patients and families at risk of either presbyacusis and/or mild or subjective cognitive impairment. Service users were considered key stakeholders by the research team if they or family members reported mild hearing loss or mild or subjective cognitive impairment.

As the app was to be designed for those who report mild or subjective cognitive impairment, it would not have been appropriate to recruit service users with a moderate-to-severe cognitive impairment. Therefore, potential participants who already had a medical diagnosis of dementia were not considered. Decisions for stakeholder inclusion were taken by the research team to ensure that the participants were representative of the desired end user of the final app. Table 1 describes in detail the types of stakeholders recruited and when they participated. This study was approved by the West Midlands–South Birmingham Research Ethics Committee. All interviews and focus groups were carried out at Imperial College Healthcare Trust, audio-recorded using a Zoom Q8, and transcribed verbatim.

**Table 1:** Description of participating stakeholders across both cycles.

Type of stakeholder		Cycle 1 (n=9)			Cycle 2 (n=14)				Total (n=23)^a^
		Interview (n=4)	Focus group 1 (n=5)	Focus group 1 (n=5)		Focus group 2 (n=5)	Focus group 3 (n=4)		
**Service user**		3	N/A^b^	5		3	N/A	11	
	Audiology	2	N/A	5		2	N/A	9	
	Cognitive disorder	1^c^	N/A	N/A		1^c^	N/A	2	
Spouse		1^d^	N/A	N/A		1^d^	N/A	2	
Volunteer		N/A	N/A	N/A		1^e^	N/A	1	
**Clinician**		N/A	4	N/A		N/A	3	7	
	Audiologist	N/A	3	N/A		N/A	3	6	
	Older adult psychiatrist	N/A	1	N/A		N/A	N/A	1	
**Researcher**		N/A	1	N/A		N/A	1	2	
	Dementia research nurse	N/A	1	N/A		N/A	N/A	1	
	PhD researcher	N/A	N/A	N/A		N/A	1	1	

^a^A total of 23 participants participated across 2 cycles. Five stakeholders participated in both cycles.

^b^N/A: not applicable.

^c^This participant is primarily a service user of the cognitive disorders’ clinic but has also used audiology services.

^d^This participant was recruited as a spouse of a service user but had also used audiology services for themselves.

^e^This participant is a volunteer in audiology but also has a hearing loss.

### Data Collection

#### Cycle 1: Identifying Current Climate

The aim of the first cycle of data collection was to first gather knowledge about the potential facilitators and barriers to designing a novel auditory-cognitive training app. To maximize accessibility of the data, the first round of data collection consisted of 2 paired semistructured interviews for service users and a focus group of 5 professional stakeholders, which were 45 min each and 60 min, respectively. This division of the stakeholder groups was beneficial for various reasons. The use of the focus group allowed discussion of professional opinions and fostered further collective thinking. Interviewing service users in pairs allowed the interviewer to explore personal experiences and views in depth by comparing and contrasting. The topic guides used for both the interviews and the focus group are available in the Multimedia Appendix 1. The exploratory nature of cycle 1 allowed the data collected to be analyzed and used in conjunction with the literature base to design the first version of the app. Further evaluation of this first version was then performed in cycle 2.

#### Cycle 2: Exploring Specific Requirements and Needs for Collaborative Design

The purpose of including a second cycle was to demonstrate the initial version of the app and to stimulate participants to further think about the specific needs and requirements of the app. To achieve the objectives of cycle 2 and answer a more specific research question about the app design, an edited topic guide was used for both the professional and nonprofessional focus groups, which were approximately 60 min in duration. The topic guides can be found in Multimedia Appendix 2.

**Figure 1:**
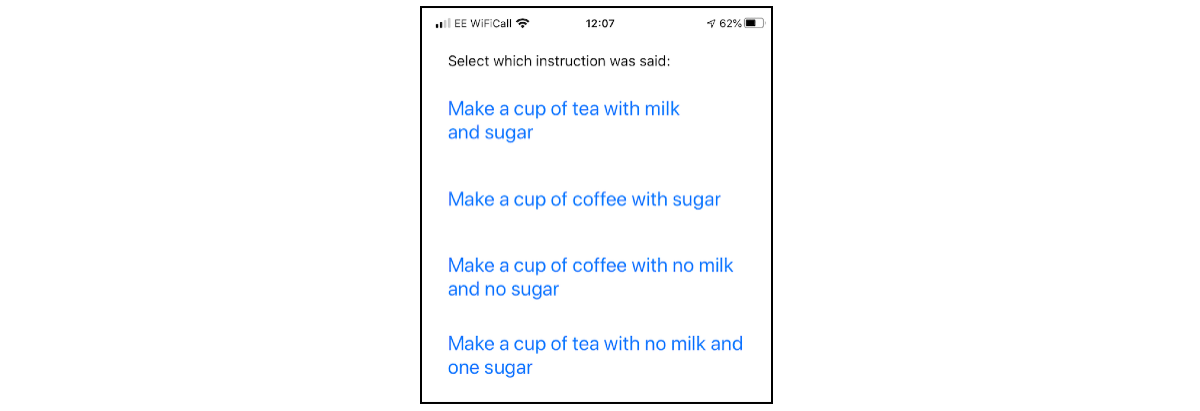
Screen where the user chooses the instruction that was heard.

**Figure 2:**
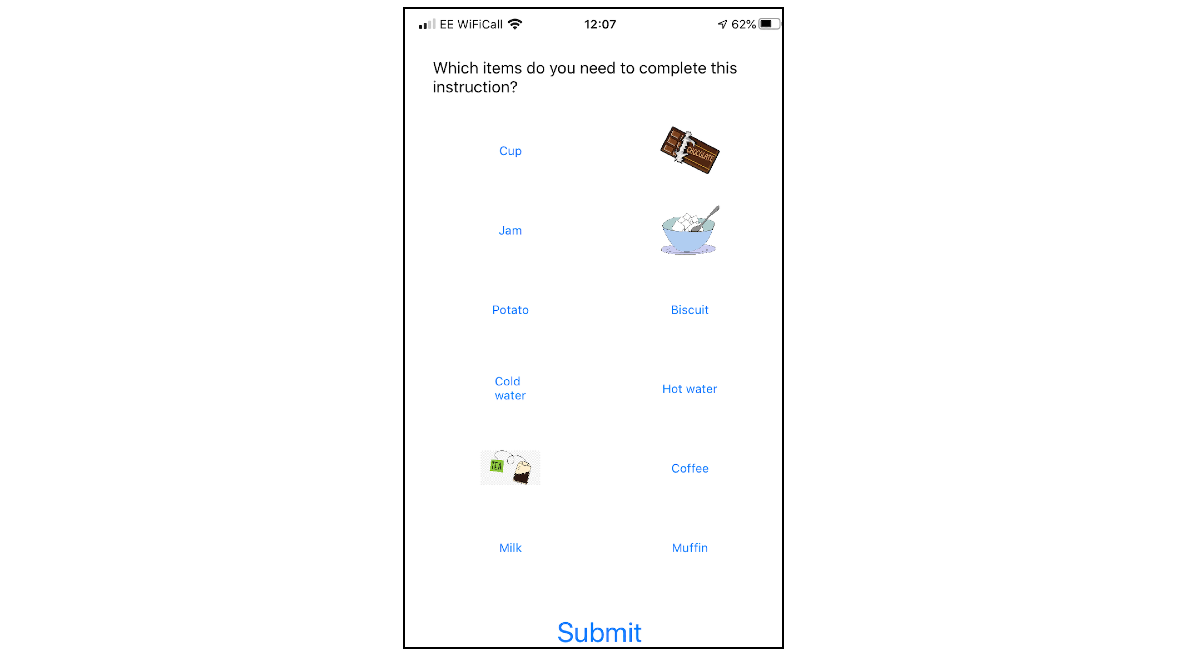
Screen where the user chooses the items that are needed to execute the audio instruction.

**Figure 3:**
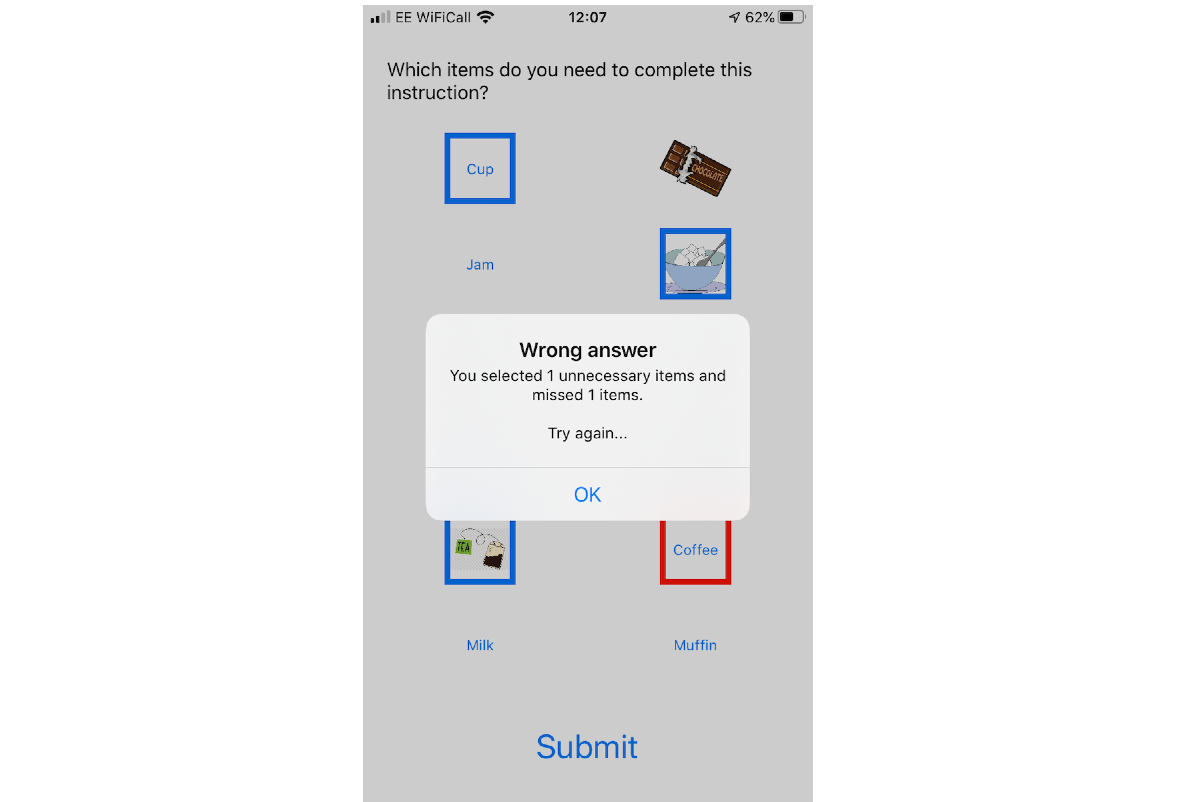
Example of incorrect answers submitted.

Two service users, 1 spouse, and 2 professional participants who wished to continue into cycle 2 participated alongside 9 new participants who did not have any prior involvement or knowledge of the app. A basic prototype of the app, using a coffee-shop scenario, was demonstrated with an iPad and the player was asked to listen to an order placed in the coffee shop, choose the correct customer order that was heard, and then choose the correct items from a list of 8 images/words to make up that specific order. The audio was originally played at a low signal-to-noise ratio, which the player could improve by 2 dB at a time by replaying the order before moving on to choosing what had been heard from a 4-option list. Screenshots from the prototype demonstrated in cycle 2 are shown in Figures 1 to 3. At the time of writing, the app is still in development and is not freely available.

**Figure 4:**
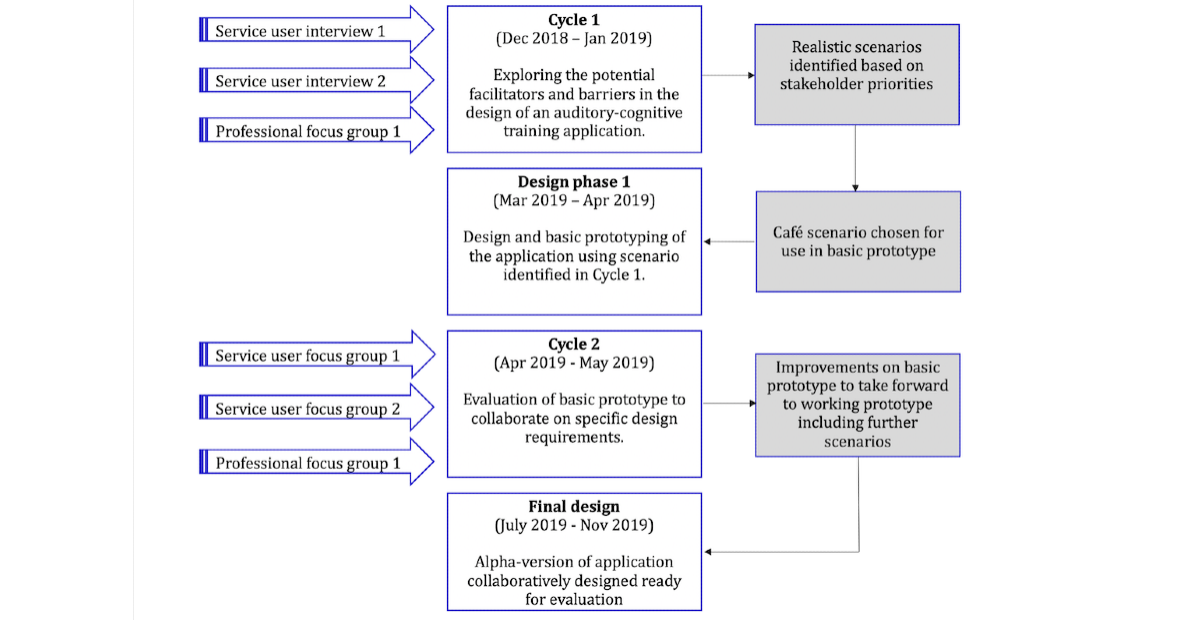
Summary of overall data collection period including design phases, start-stop for data collection, and stakeholder type participation.

A summary of the data collection, including the start and stop dates, the duration of each cycle, and stakeholder participation is shown in Figure 4.

### Data Processing

Audio recordings from the interviews and focus groups were transferred immediately to an encrypted PC and deleted from the audio recorder. The recordings were transcribed by the lead author in cycle 1 and by medical students who had received prior training in cycle 2. The transcriptions were subsequently coded by the lead author in cycle 1 and medical students in cycle 2 and stored in Microsoft Excel. To verify the integrity of the data, each cycle was secondary coded by either the lead author or medical students. All excerpts from the transcriptions were anonymized using the participant number that identified each participant as either a service user or a professional.

### Data Analysis

A thematic analysis approach [13] was used to identify themes from the data related to the facilitators, barriers, and needs of the stakeholders in developing a new auditory-training gaming app. This process involved the lead author and trained students identifying themes from the codes.

## Results

### Overview

Six themes were identified from the focus groups in relation to the needs, barriers, and facilitators for developing an auditory-cognitive app that would be useful, fun, and accessible. The themes were (1) congruence with hobbies, (2) life gets in the way, (3) motivational challenge, (4) accessibility, (5) additive competition, and (6) realism.

### Congruence With Hobbies

Throughout the focus groups, service users commented particularly on what would continue to motivate them to play the game over an extended period of time, rather than a one-off use. At first, it appeared to be different styles or themes of games that motivated them, such as word games or web-based chess. However, further discussion by 2 service users who did not regularly play mobile games led to the consensus that if the theme of the game was an extension of an enjoyable hobby, then this would heavily facilitate not just initial interest, but extended and continued playing time:

I mean this game is educational but you want to make it fun as well, fun at the same time.

SU9

For example, if you had one about art?

SU8

Yes, I'd use it.

SU9

You'd be at it all day!

SU8

### Life Gets in the Way

Although both service users and clinicians agreed that the premise of the app was good, they noted finding the time to use the app as a potential barrier. It was suggested that the user, for practical purposes, would need to be at home in a quiet space to be able to use headphones and concentrate. Others suggested that busy lives meant that other responsibilities, such as taking care of grandchildren or house chores, took priority over self-care. This feeling of being too busy to use the app has parallels with reactive or passive health care, such as using hearing aids after a hearing loss has been diagnosed, as opposed to a preventative or active approach, whereby spending a small amount of time each day may, in fact, benefit in the longer term:

I find that I don't pick up my iPad and read the paper anymore, I didn't realise I didn't do it. But then when I went to my iPad it had no battery and I didn't care, so it was kind of like oh I've stopped reading the paper, but I didn't really notice.

SU10

I'm also quite involved in the church and the grandchildren. So, I think when you are saying as to what might prevent you from doing these other pleasurable things, then it would be other equally pleasurable things that one has to do.

SU8

Participants suggested using notifications and reminders within the app to remind users that they were overdue for a training session or to use commuting time on the underground or train as an opportunity to play. Interestingly, in the professional group, there was a misalignment in views regarding whether people in an older age group would engage with smartphones and headphones while traveling:

I don't think I've seen anyone like in their sixties even [using a smartphone?].

P1

I have.

P2

Have you been on the tube?! I think they do!

P3

I could probably play it on the bus or something you know, when you are travelling, something to just fill time.

SU7

Yes, on the tube.

SU10

### Motivational Challenge

Despite the need to set aside some time to play each day, one main facilitator identified by both the service user and clinician groups was that the app should provide the correct amount of motivation and an element of challenge to ensure it was fun, useful, and enticed the user back to play. The motivation did not necessarily need to come from improving one’s own prowess. The idea of altruism as a motivator was discussed. It was suggested that if a person were aware that playing the app would contribute to research knowledge on dementia, they would be much more likely to play it though it may not necessarily gratify them personally in the short term. The professional group suggested that using multiple scenarios would increase the relevance to challenges faced by people with impaired hearing and, therefore, increase the motivation to improve in all situations:

For me I would be more encouraged if I knew it was paying back into research. If I knew that somebody thought it was good for me too. If I was playing it just for the sake of playing it then I would be playing it for nothing. But if I'm playing it and I'm contributing then I can pretend I'm contributing even if I'm just playing for myself.

SU10

It would be good if they can select what do you want to train. I had the problem the other day speaking with my friend at the cafe...Maybe I'll give it a go, yes today I'll play the cafe.

P19

It's motivation as well because if you do terribly at the one in the cafe but you're doing fantastic at all the others. I am going to the do that cafe one again, I am going to smash the cafe one today.

P1

### Accessibility

One clear need highlighted from both cycles was that the app needed to be accessible to people of all ages, catering to those with visual or audio impairments and available for playing on appropriate platforms. Appropriate screen resolutions, font sizes, images, and colors were design parameters identified as important:

I have been testing patients as well, elderly patients for my study and I use an app on the iPad as well as computer tasks and they tolerate it very well. They never complain when I say let’s switch it to the iPad, actually they like the one on the iPad more.

P19

I think most people are going to play for that age on a phone or an iPad. I think it would need to go across both platforms because what is it 25% Apple, 75% Android?

SU3

If I could do it via the computer... simply I'm used to using the computer.

SU8

I think the size is relevant actually, that tiny screen it [of a smartphone] it's not quite the same as if you were looking on the screen.

SU9

### Addictive Competition

Comparing the premise of this app with those of other successful games that the participants played resulted in an agreement to the reason why people went back to playing certain games repeatedly and over a long period of time. The app or games that were the most successful were addictive, not only in terms of the aims of the games themselves but also in terms of the competitive nature of moving through levels to beat a family member, partner, or friend. Having a shared platform to engage in a healthy competition was seen as a driver for playing an app. Scores, rewards, and trophies were all seen as optional extras that would provide extra facilitation in prolonged and repetitive play:

Maybe I could compare this with my husband or my friends and then I would know they were able to do it like three tones before me, so maybe I am actually a bit worse.

P19

Another way that I've mentioned might be to pair up with a relative or have some kind of competitive nature you know in the household.

P5

My mother-in-law could see my scores if that was of interest to her.

SU10

Gives you more motivation I think, if you're in a competitive nature.

SU13

### Realism

The app in cycle 2 was demonstrated using a coffee-shop scenario, which received positive feedback from all participants as it involved a real-world environment in which it was likely that a person may have difficulty hearing speech. All participants agreed that it would be most appropriate to use *real-life* scenarios in the game, instead of complete gamification. It was suggested that using realistic scenarios would make the app more useful and the skills built in the game more transferable. It was suggested that using realistic tasks would also make the game more appealing to an older person, as it made it feel less like a game. The clinician group felt that using these scenarios would also make it easier for them to recommend the game and also to use the game to obtain feedback about specific situations in which the person had specific difficulties. By being realistic, the game would also tie in with the theme of using hobbies as scenarios:

It has to cover areas that a person like myself would find it very difficult to hear, like for instance I said the gym, but also airports they can be a nightmare as well, you know I still have to travel even if I'm deaf.

SU13

Particularly if they are already isolated and they're already staying at home and they're sort of scared of going outside, it's a nice way to bring outside in, so they can build up their experience in other situations without actually having to get there.

P3

And it's an element of control that they're taking over their situation and so that it'll give me some confidence you know I'm doing something about it. Makes you feel good.

P1

### Summary of Results

The summary of results has been provided in Table 2.

**Table 2:** Summary of findings in relation to the research question.

Theme	Facilitators	Barriers	Needs
Congruence with hobbies	Initiate and maintain interest over time to allow repeated play Concentrate on being educational and enjoyable	Limitations in terms of the number of preprogrammed scenarios catering to all hobbies	Relate to common enjoyable hobbies for the intended user groups
Life gets in the way	Promote usage of the app during unavoidable daily tasks, for example, commuting	Incorporating the app into busy daily lives Reliance on passive health care models	App to send notifications and reminders when the user is overdue for a training session. Allow offline play, for example, when commuting
Motivational challenge	Promote altruism to contribute to research	App not offering the right level or type of challenge leading to lack of repeated playing and training.	Level of difficulty to be challenging enough to entice repeated play Multiple scenarios relevant to difficult hearing situations
Accessibility	Design considerations, for example, use of colors, font sizes, and images	Smaller screens on smartphones Inappropriate screen resolution for each device	Accessible to all ages Available on multiple platforms and devices, including PCs
Addictive competition	Option to share progress with family and friends to encourage competition	Not a driver for playing for people who are not of a competitive nature	Include daily high scores that are comparable with friends or self across time
Realism	Skills honed in the app would be more transferable More likely to recommend to friends	May prefer more realistic graphics rather than taking a gamified approach	Relatable to real-life environments where hearing is difficult

## Discussion

### Principal Findings

This study aimed to engage relevant stakeholders from the worlds of audiology and cognitive disorders to collaborate in the design and development of an auditory-cognitive training game app. Stakeholders were recruited and engaged in 2 cycles of semistructured paired interviews and focus groups to understand the facilitators and barriers in producing such an app and to elicit specific design requirements for the app in addition to the existing literature.

### Facilitators

A popular choice for facilitating a new gaming app was to provide a high level of addictive competition for the user. The results demonstrate that this can be achieved in a number of ways, including rewards, achievements, and competitive play with family and friends. This has parallels with the findings of Talaei-Khoei and Daniel [12], who found that their participants were motivated to improve their memory age as they experienced with a sense of achievement and reward. This study also found that participants wanted an extra level of socialization within the app through a virtual competition with friends to share scores and achievements.

This sharing of information was also addressed to ensure that the app was motivationally challenging enough to encourage them back to play. A particularly interesting finding was that the motivation to play the app was not necessarily to improve one's own skills, or for personal gain, but to provide data altruistically to a research database on a topic such as dementia. This has parallels with the popularity of *Sea Hero Quest* developed by Deutsche Telekom, which has been downloaded by over 4.3 million players [14]. *Sea Hero Quest* is an app developed to collect large data sets on how navigational cognition changes over the human life span. Collecting data through gameplay has provided data that would have taken a long time to obtain through standard dementia research practices.

### Barriers

One of the themes that was perceived as a barrier to produce a successful app for high adoption was that other life activities would get in the way of using the app on a regular basis, as it would require a quiet space to concentrate. Participants gave examples of other activities that required their attention and efforts that were placed above auditory-cognitive training, such as household chores. The low level of importance placed on maintaining cognitive reserve in the light of other daily activities by participants is in contrast to the theory of Weinstein [15], who suggests that building cognitive resilience is of utmost importance in the window of opportunity that is midlife. It is critical to engage the cognitive reserve in midlife to allow the brain to cope better with damage in later life.

The findings from this study demonstrate that even with this knowledge, changing health behaviors is challenging and often unsuccessful [16]. It is therefore critical to adopt the proposals of Talaei-Khoei and Daniel [12] and employ qualitative methods, as in this study, to focus specifically on why end users would find a training game to be useful and adopt it.

One potential barrier to using hobbies as a motivator is the effect of apathy on motivation. Apathy is a major neuropsychiatric symptom in dementia and is sometimes observed in patients with MCI [17]. Individuals with clinical apathy would be less likely to be motivated by the type of training described. However, it should be noted that the intended user group is specifically focused on individuals with subjective cognitive impairment and MCI, who have a much lower incidence of apathy than those with more severe cognitive impairment [18].

### Needs

One of the specific requirements that was elicited from the discussion was to ensure that the app was accessible to older adults, who may be unfamiliar with using tablets or smartphones to access apps. This is also a potential barrier noted in the general gaming literature. However, according to Vallejo et al [11], no usability problems were reported for participants without previous computer experience when using a joystick or touchscreen.

Interestingly, participants felt that making the scenarios less gamified, more realistic, and more related to daily living would be more useful, transferable, and more appealing to older adults. The results showed that if the scenarios were congruent with or an extension of an enjoyable hobby for the end user, this would increase the level of interest, fun, and ultimately adoption. This finding could explain why laboratory-style auditory training programs, such as those evaluated by Ferguson and Henshaw [19], have failed to extrapolate on-task learning to off-task daily activities. As suggested by Anguera et al [9], their training game was successful as it was being delivered outside of the laboratory environment and because of its custom design. Similarly, the reason Manera et al [10] found large variations in playing time in their app based on cooking may be due to the lack of engagement and interest from some of their users. Therefore, the use of multiple common scenarios based on daily activities and a custom scenario based on a hobby might, in fact, increase the adoption and success rate outside of the app.

### Limitations

The use of a small sample size is more common in participatory design, as it is about the rich quality of data rather than the quantity. Demographically, the age range and the use of hearing aids were skewed from the desired end user group. However, it allowed exploration of using this type of app as a supplement to hearing aid provision in more severe hearing losses in the future. As this app is in its infancy and is yet to be evaluated for its effectiveness, there is potential to use the app in other, more hearing-impaired populations. However, for the scope of this study, involving those with varying hearing loss severities would introduce a confounding variable during assessment if using the app does indeed improve unaided speech listening in noise.

### Lessons Learned

#### Stakeholder Recruitment

The inclusion of nonclinical stakeholders that already have existing relationships can enhance data collection. In both cycles, the stakeholders included spouses and friends. This extended the depth of data collection around more sensitive questions, such as thoughts and feelings about developing cognitive impairment and current cognitive performance. Stakeholders were more comfortable discussing these issues with someone they already knew as opposed to the interviewer. This was observed in a design workshop with aphasia patients [20]. The author concluded that using a relative is essential in fostering a *communication culture*, which gives the stakeholder with the condition confidence to express and verbalize his or her thoughts and feelings.

It was also useful for stimulating further discussion, as the stakeholders had more background information about one another in comparison with the interviewer. Stakeholders were able to ask further appropriate probing questions when discussing content. This was evident when discussing possible scenarios for the gaming levels, as one stakeholder was able to talk more to his or her spouse about his or her enjoyment of art galleries and bring this idea to the discussion.

When holding clinical stakeholder focus groups, a multidisciplinary discussion should be used not only to uncover shared thinking that provides useful data for answering the research question but also to take the use further into wider clinical practice outside of the app design. In this instance, mixing clinicians from audiology, psychiatry, and cognitive disorders research brought together specialists who do not usually meet but share common patient groups and challenges. This allowed clinicians time away from their individual departments to discuss ways in which they could support each other to improve the care of patients who may unknowingly access each other’s services; for example, implementing the use of a hearing screening pathway for patients referred for cognitive assessment to trigger a referral for audiology assessment and facilitate communication in cognitive assessments. Where possible, clinical stakeholders should include those with a range of experience from the newly qualified to the consultant level, to tap into both new learning and wealth of experience. Consideration should also be given to include geographical variance to allow for deviances in service delivery away from national guidelines. Woods et al [21] used co-design to develop a mobile health app in the area of cardiac health and concluded that using participatory design within a health care delivery setting with multiple clinicians improved patient-centered care. Using participatory design with multidisciplinary stakeholders can facilitate a wider and unforeseen positive impact across service delivery, both locally and nationally.

When focusing on designing an app to be used in a preclinical symptomatic population, it is prudent to recruit from multiple sources outside of the standard clinical settings, such as hospitals and GP clinics. Groups in the community, such as clubs, neighborhood associations, and religious groups, should be targeted as potential sources of recruitment as they are likely to include stakeholders that may have symptoms that are not severe enough to seek clinical intervention and therefore do not frequent clinical settings such as audiology or cognitive disorder clinics.

It may also be useful to include stakeholders from a wider pool that, although may be less relevant to prospective end users, can offer ideas for future implementation of the app. Stakeholders that have already experienced a condition can provide data on past experiences. These stakeholders are also useful in patient and public involvement activities before data collection begin and can give advice on research question development, advertising materials, and focus group questionnaire design.

#### Developing Content and Gameplay

When asking stakeholders to contribute to designing content for a new game or task that requires an element of training or behavior change, it is important to begin the discussion by asking the stakeholders about hobbies or activities that they already enjoy participating in. Particular focus should be given to why they enjoy them and what stimulates them to participate regularly in that particular activity. For example, in cycle 2, web-based chess and web-based crosswords were introduced as enjoyable platforms for distraction, competition, and accessibility at all times of the day.

The reasons behind successful adoption and enjoyment of other apps should be understood and consequently integrated into tasks for the new game in conjunction with recommendations from the literature specifying special attention to customization and individualization. This was evident when discussing design for different scenarios. Stakeholders foresaw that they were more likely to regularly use the app if the scenario was individualized to an environment that they associated with enjoyment or relaxation, such as an exercise class or an art gallery. In addition, they were also likely to use it if it was customized to a situation in which they found it difficult to communicate in reality and would want extra practice virtually. Examples include cafés, airports, and train stations. Similarly, Jessen et al [22] used this approach while researching participatory design frameworks for a self-management app in a chronic disease population. They used common enjoyable games such as Super Mario, Crosswords, and Monopoly as a vehicle to elicit further thoughts for discussion on the concept of creating their design.

### Further Development of the App

To overcome these barriers and incorporate the design needs from the findings, within the scope of the project, the app will include:

- The option to deliver daily notifications to remind the user to take some time to play the app
- The inclusion of scores and comparisons to daily or weekly high scores
- A redesign of the visual representation to use only images as opposed to a mixture of words and images
- Colored boxes (green=correct; red=incorrect) that will appear around the images when selected to notify the user of correct and incorrect selections during repeated attempts
- A range of 6 realistic scenarios reported by people with a hearing impairment as challenging
- Inclusion of a customized scenario for participants evaluating the app that they consider relevant to them
- Allowing offline play so that users will be able to play the app during other daily activities, for example, commuting

### Conclusions

Using a participatory approach in conjunction with the literature base when designing a novel app ensures that the final product is useful, fun, and accessible to the intended user group. Both cycles of this project have demonstrated that an app that can provide training for both auditory and cognitive performance in a way that would motivate users to regularly play it would be welcomed as an alternative to hearing aids, and as a fun activity, and it will be used if it could keep the brain active and healthy. The idea of completing an active, preventative task still does not carry enough weight to drive people to use it and, therefore, would require other competitive and reward elements to overcome the barrier of having enough time to use it. The results of this study will now be used to finalize the app design and complete a randomized controlled study to evaluate the effectiveness of using the app on speech-in-noise, cognitive ability, and quality of life, in addition to usability evaluation.

## Appendix

Multimedia Appendix 1 Topic guides used in cycle 1.

Multimedia Appendix 2 Topic guides used in cycle 2.

## Abbreviations

AD: Alzheimer disease
GP: general practitioner
MCI: mild cognitive impairment
WHO: World Health Organization
