# Supplementary material for: A Novel Auditory-Cognitive Training App for Delaying or Preventing the Onset of Dementia: Participatory Design With Stakeholders
Source: JMIR Hum Factors. 2020 Sep 30;7(3):e19880. doi: 10.2196/19880 (PMC7557448; doi:10.2196/19880)
Supplement: Multimedia Appendix 1 [file humanfactors_v7i3e19880_app1.docx]

**Topic Guide – Round 1 Professionals**

Before the focus group starts:

- Check all participants have read and understood the participant information sheet and signed the consent form.
- Remind them that participation is voluntary and that they can withdraw at any time.

Explain the ground rules for the session:

- This is a respectful discussion and everyone’s opinions are valid, so it is important to not interrupt each other or talk over one another.
- Anything said in the focus group is to remain confidential. The session will be audio recorded, however participants will not be identifiable as participant numbers will be used.
- Participants are free to ask questions or take breaks at any time they wish.

Start of focus group:

My name is Emily and I will be facilitating the discussion. Please can everyone introduce themselves to the group.

I will give brief overview of the project and what it is that we will be discussing today.

The research project is looking at the relationship between aged-related hearing loss and developing problems with memory and attention, which may then lead onto dementia. To do so, the project is looking to design and develop a brand new application that can be played on mobile phones or tablets that can provide training for hearing, memory and attention. This project is part of my PhD and I want to explore and discuss views and attitudes towards using an application or game and how best to design the game to make sure it is appropriate and fun for the users.

I have some questions related to this idea which will help guide our discussion. I am interested in finding out what you honestly think.

Warm-Up questions:

- How important is your physical health to you and your patients?
- How important is your mental health to you and your patients?
  - Is this any different to physical health?
- Where would you suggest patients find advice on their physical and mental health?

Section 1 – Hearing and Dementia

- How important is it for your patients to have good hearing and a good memory?
- Are you aware of hearing loss and dementia being linked?
  - Are your patients aware?
  - Would you discuss this link with them?
- Do you think we encourage our patients to look after their hearing and memory well enough?
- If your patients had concerns over their hearing or their memory, what would you advise them to do?
  - Where would you tell them to look for information?
  - Would you communicate this to other medical professionals?
- Do you currently recommend any learning or training material for your patients’ hearing and memory?
  - Websites?
  - Organisations?
  - Apps?
  - Groups?

Section 2 – Using Technology

- Do you own a smartphone or tablet
  - If yes, why?
  - If no, why not?
- How do these devices help or hinder us in our daily lives?
- Do you think you utilise technology in your clinical practice?
  - Are you aware of anything particular that you feel is useful?
  - Anything that isn’t useful? If so why?
- How useful could apps be for healthcare in the future?
  - Is there anything in particular you think would be useful?
  - What do you foresee as barriers to your patients accessing and using an app?
  - What do you foresee as potential gains to your patients accessing and using an app?

Section 3 – Designing the Application

Action – Demonstrate ‘Sea Hero Quest’ app and explain the purpose and popularity.

- What do you think of the app?
- What do you like and dislike about the app?
- What would make a good app for people aged 50 and over to play?
- Thinking about hearing and memory, what tasks do you think your patients would like to complete in the app?
- What would make you want to recommend an app to your patients?
  - Would you like it to be realistic or more of a game?
- Would you prefer for the patient to use the app on their own or would you prefer they use it with other people?
  - Guide or advisor?
  - 2^nd^ player online?
  - In your clinics?
- Does anyone have any further questions or comments to make?

After the group has finished:

- Thank everyone for participating and volunteering their time.
- Explain what will happen next e.g transcription, formalising a report to the developer, developer to start of first prototype.
- Explain when the next focus group will be and check participants are happy to continue and attend the next group.
- Remain in the room in case participants would like to discuss any particular concerns. Highlight the leaflets regarding hearing loss, dementia and how to seek help if concerned.
- Ensure participants leave with a copy of the participant information sheet and consent form.

**Topic Guide – Round 1 Service Users**

Before the focus group starts:

- Check all participants have read and understood the participant information sheet and signed the consent form.
- Remind them that participation is voluntary and that they can withdraw at any time.

Explain the ground rules for the session:

- This is a respectful discussion and everyone’s opinions are valid, so it is important to not interrupt each other or talk over one another.
- Anything said in the focus group is to remain confidential. The session will be audio recorded, however participants will not be identifiable as participant numbers will be used.
- Participants are free to ask questions or take breaks at any time they wish.

Start of focus group:

My name is Emily and I will be facilitating the discussion. Please can everyone introduce themselves to the group.

I will give brief overview of the project and what it is that we will be discussing today.

The research project is looking at the relationship between aged-related hearing loss and developing problems with memory and attention, which may then lead onto dementia. To do so, the project is looking to design and develop a brand new application that can be played on mobile phones or tablets that can provide training for hearing, memory and attention. This project is part of my PhD and I want to explore and discuss views and attitudes towards using an application or game and how best to design the game to make sure it is appropriate and fun for the users.

I have some questions related to this idea which will help guide our discussion. I am interested in finding out what you honestly think.

Warm-Up questions:

- How important is your physical health to you?
- How important is your mental health to you?
  - Is this any different to your physical health?
- How often would you see your GP for advice?
  - Would you seek advice or information elsewhere? Where?

Section 1 – Hearing and Dementia

- How important is your hearing to you?
- Are you aware of hearing loss and dementia being linked?
- Do you think we look after our hearing and memory well enough?
- If you had concerns over your hearing or your memory, what would you do?
  - Where would you look for information?
  - Who would you tell?

Section 2 – Using Technology

- Do you own a smartphone or tablet
  - If yes, why?
  - If no, why not?
- How do these devices help or hinder us in our daily lives?
- How useful could apps be for healthcare in the future?

Section 3 – Designing the Application

Action – Demonstrate ‘Sea Hero Quest’ app and explain the purpose and popularity.

- What do you think of the app?
- What do you like and dislike about the app?
- What would make a good app for people aged 50 and over to play?
- Thinking about hearing and memory, what tasks would you like to complete in the app?
- What would make you want to use the app every day?
  - Would you like it to be realistic or more of a game?
- Would you prefer to use the app on your own or would you prefer to use it with other people?
  - Guide or advisor?
  - 2^nd^ player online?
- Does anyone have any further questions or comments to make?

After the group has finished:

- Thank everyone for participating and volunteering their time.
- Explain what will happen next e.g transcription, formalising a report to the developer, developer to start of first prototype.
- Explain when the next focus group will be and check participants are happy to continue and attend the next group.
- Remain in the room in case participants would like to discuss any particular concerns. Highlight the leaflets regarding hearing loss, dementia and how to seek help if concerned.
- Ensure participants leave with a copy of the participant information sheet and consent form.
