# Supplementary material for: A Novel Auditory-Cognitive Training App for Delaying or Preventing the Onset of Dementia: Participatory Design With Stakeholders
Source: JMIR Hum Factors. 2020 Sep 30;7(3):e19880. doi: 10.2196/19880 (PMC7557448; doi:10.2196/19880)
Supplement: Multimedia Appendix 2 [file humanfactors_v7i3e19880_app2.docx]

**Round 2 - Topic Guide – Professionals**

Before the focus group starts:

- Check all participants have read and understood the participant information sheet and signed the consent form.
- Remind them that participation is voluntary and that they can withdraw at any time.

Explain the ground rules for the session:

- This is a respectful discussion and everyone’s opinions are valid, so it is important to not interrupt each other or talk over one another.
- Anything said in the focus group is to remain confidential. The session will be audio recorded, however participants will not be identifiable as participant numbers will be used.
- Participants are free to ask questions or take breaks at any time they wish.

Start of focus group:

My name is Emily and I will be facilitating the discussion. Please can everyone introduce themselves to the group.

I will give brief overview of the project and what it is that we will be discussing today.

The research project is looking at the relationship between aged-related hearing loss and developing problems with memory and attention, which may then lead onto dementia. To do so, the project is looking to design and develop a brand new application that can be played on mobile phones or tablets that can provide training for hearing, memory and attention. This project is part of my PhD and I want to explore and discuss views and attitudes towards using an application or game and how best to design the game to make sure it is appropriate and fun for the users.

I have some questions related to this idea which will help guide our discussion. I am interested in finding out what you honestly think.

Using digital games:

- Do you think your patients ever use digital games or gaming apps?
  - How often do you use digital games?
  - What games do you think they play?
  - What do you think they like about these games?
  - What do you think they dislike about these games?
  - What motivates them to play these games?
  - Do you think your patients use apps?
  - Which apps do you think your patients may use the most?
  - Do you currently recommend any auditory or cognitive training games to your patients?
    - Why? Which ones? How do patients react?
    - Why not?

Looking at the alpha version of the app:

Context:

- Are the situations shown in the app relevant to the daily lives of people with hearing loss?
- What sounds would make the environment sound like the real place?
- What other situations do you think would work well as a game?
  - Why?

Concept:

- Do you think these sorts of challenges in the game would provide the right level of difficulty?
- Would the game help to increase your patients’ confidence in their hearing in noise OR their short-term memory?
- Would you prefer your patients to play the game alone of under supervision in the clinic?
- Do you think the scenario e.g café should change?
- Do you think patients would prefer a customised scenario?

Playability:

- Would you use this game? Or recommend it to your patients?
  - How often would you recommend they play this game?
  - What would motivate your patients to play it?
  - What would prevent your patients from playing it?
  - Do you think nudge notifications would work?
  - What sort of incentives would encourage you or your patients to play the game?
  - Would it be helpful for you to receive information on the scoring for your patients?
  - Could you see this being a tool to be used in patient’s rehabilitation or counselling?
- What device would you or your patients prefer to play this game on?
- How do you think the instructions should be given?
- Do you prefer pictures or words for the buttons?

**Round 2 - Topic Guide – Service Users/Volunteers**

Before the focus group starts:

- Check all participants have read and understood the participant information sheet and signed the consent form.
- Remind them that participation is voluntary and that they can withdraw at any time.

Explain the ground rules for the session:

- This is a respectful discussion and everyone’s opinions are valid, so it is important to not interrupt each other or talk over one another.
- Anything said in the focus group is to remain confidential. The session will be audio recorded, however participants will not be identifiable as participant numbers will be used.
- Participants are free to ask questions or take breaks at any time they wish.

Start of focus group:

My name is Emily and I will be facilitating the discussion. Please can everyone introduce themselves to the group.

I will give brief overview of the project and what it is that we will be discussing today.

The research project is looking at the relationship between aged-related hearing loss and developing problems with memory and attention, which may then lead onto dementia. To do so, the project is looking to design and develop a brand new application that can be played on mobile phones or tablets that can provide training for hearing, memory and attention. This project is part of my PhD and I want to explore and discuss views and attitudes towards using an application or game and how best to design the game to make sure it is appropriate and fun for the users.

I have some questions related to this idea which will help guide our discussion. I am interested in finding out what you honestly think.

Warm-up Questions:

- What are your main hobbies or interests?
- What motivates you to take part in these hobbies?
- What prevents you from taking part in these hobbies?
- Do you prefer physical or mental hobbies?
  - Why?

Using digital games:

- Have you ever used digital games or gaming apps before?
  - How often do you use digital games?
  - What games do you play?
  - Do you play traditional games? E.g chess, board games, jigsaws?
  - What do you like about these games?
    - What motivates you to play these games?
    - Do you pay for apps/games?
    - How do you decide which apps to download?
  - What do you dislike about these games?
    - What prevents you from playing these games?
    - What prevents you from playing these games for a long period of time?
  - Who do you play digital games with?

Looking at the alpha version of the app:

Context:

- Are the situations shown in the app relevant to the daily lives of people with hearing loss?
- What sound would make the environment sound like the real place?
- What other situations do you think would work well as a game?

Concept:

- Do you think these sorts of challenges in the game would provide the right level of difficulty?
- Would the game help to increase your confidence with your hearing in noise OR your short-term memory?
- Would you prefer to play the game alone of under supervision in the clinic?
- Would you like the scenario e.g café to change?
- Would you prefer a customised scenario?

Playability:

- Would you use this game?
  - How often would you play this game?
  - What time of day would you play the game?
  - What would motivate you to play it?
  - Would you like to receive nudges?
  - What sort of incentives would encourage you to play the game?
  - What would motivate you to play this game for a long period of time?
  - Would you like to play this game with other people?
- What device would you prefer to play this game on?
  - Would you play this with headphones?
- How would you like to receive the instructions?
- Do you prefer pictures or words for the buttons?
